# Reduction in STIs in an empowerment intervention programme for female sex workers in Bangalore, India: the Pragati programme

**DOI:** 10.3402/gha.v6i0.22943

**Published:** 2013-12-27

**Authors:** Dennis Souverein, Sjoerd M. Euser, Rajendra Ramaiah, Pushpalatha Rama Narayana Gowda, Chandra Shekhar Gowda, Diana C. Grootendorst, Snehal Barot, Françoise Jenniskens, Sunil Kumar, Shiv Kumar, Jeroen W. Den Boer

**Affiliations:** 1 Department of Epidemiology, Regional Public Health Laboratory Kennemerland, Haarlem, The Netherlands; 2 Swasti A Health Resource Center, Bangalore, India; 3 Swathi Mahila Sangha, Bangalore, India; 4 Françoise Jenniskens HIV and Health Advice, Haarlem, The Netherlands; 5 Karnataka Health Promotion Trust, Bangalore, India

**Keywords:** sexual health, prevention, India, syndromic management, empowerment

## Abstract

**Background/Objective:**

The Pragati programme is an on-going empowerment programme for female sex workers (FSWs) working and living in Bangalore, India. Pragati aims to reduce transmission of HIV and sexually transmitted infections (STIs) among FSWs. This study describes the STI incidence rate, contact rate, and condom use during follow-up years.

**Design:**

Between April 2005 and November 2010, 20,330 FSWs participated in the programme. Outcome measures were programme exposure (number of contacts per person-year), STI incidence rate, and condom use. All analyses were stratified by year of follow-up. STIs were diagnosed by syndromic case management in either programme or referral clinics. We restricted our analyses to the period between April 2005 and July 2008 (when the majority of STIs were diagnosed in programme clinics), in order to minimise the possible influence of differences in STI diagnosis between clinic types.

**Results:**

Results showed a significant increase of programme exposure (*p*-value for trend < 0.001) and a significant decrease in the STI incidence rate (*p*-value for trend < 0.001) over the follow-up time (between April 2005 and July 2008). Reported condom use at last paid sex increased from 77.6% in year 1 to 100% in year 4 of follow-up (*p*-value for trend < 0.001).

**Conclusion:**

Our data seem to suggest that the Pragati programme had a positive effect on the STI incidence rate and condom use, possibly as a result of increased programme exposure. We recommend for future studies to invest more in the study design, type of data collection, and recording mechanisms before starting with an intervention. Incorporation of empowerment strategies as an approach in HIV prevention programmes can have a beneficial effect on the lives and livelihoods of FSWs.

India has one of the highest numbers of people living with HIV (PLHIV) in the world, with an estimated 2.7 million in 2002 (1). Although this number decreased slightly as of 2009 (2.4 million; 0.45%), it remains high among high-risk groups such as female sex workers (FSWs) (5.1%), men who have sex with men (7.4%), and injecting drug users (7.2%) (1–3).

Previous studies showed that sexually transmitted infections (STIs) increase the probability of HIV transmission two- to five-fold (4–8). Reducing the number of STIs within individuals who are at increased risk of HIV infection (e.g. by empowering FSWs to adopt safer sex) might therefore result in beneficial effects on HIV incidence (9, 10). Several interventions to reduce the HIV incidence among FSWs in India have been reported, for example, an HIV prevention programme for FSWs in Karnataka. Outcome measures were prevalence rates of HIV, syphilis, and other STIs (chlamydia and gonorrhoea) (obtained from cross-sectional laboratory-confirmed surveys). Between 2004 and 2009, reductions in all STIs were reported together with increasing condom use and programme exposure rates (11). In line with the above-mentioned study, the same outcome measures were studied in the Avahan programme, another large HIV intervention programme in India (12). Reduced prevalence rates of STIs, high programme exposure coinciding with increasing condom use, declining syphilis rates, and stabilising HIV prevalence were reported (12). Both above-mentioned studies are behavioural change interventions which address a broad range of FSW-related problems. The results of a Cochrane review by Wariki et al. are in line with previously mentioned studies and showed favourable results for behavioural interventions over single interventions in reducing STI incidence among FSWs (13). That review also showed that promotion of condom use was strongly related to STI prevalence, especially in low- and middle-income countries. In addition, the study by Wariki et al. and several other studies suggest that incorporating empowerment strategies in interventions may further improve coverage, acceptability, and compliance to interventions in relation to single interventions (e.g. only STI treatment and/or condom promotion) (13–16).

The Pragati programme is a large-scale intervention programme among FSWs in Bangalore, India (17). It uses an empowerment approach to address issues that threaten the FSW community. The hypothesis of the programme is that empowerment of FSWs enables them and the FSW community to better protect themselves from STIs (including HIV) and other health threats. The empowerment strategy in the Pragati programme includes a crisis response team, a microfinance system, a de-addiction programme, condom use promotion, STI prevention and detection (with the use of syndromic case management, or SCM), and the provision of rest places (to douche, relax, and meet peers). This so-called empowerment strategy is a holistic approach which includes all relevant factors that play an important role in the HIV epidemic affecting FSWs and the FSW community in Bangalore. The Pragati programme distinguishes itself from other conventional HIV prevention programmes with key components such as empowerment and SCM.

SCM is a tool to diagnose and treat STIs based on syndromes. If a syndrome is detected, treatment will follow immediately after the first visit, which leads to high compliance. Vaginitis, cervicitis, genital ulcers, and pelvic inflammatory disease (PID) are examples of common syndromes. Advantages of this method are (1) rapid diagnosis, (2) treatment, and (3) feasibility in low-resource settings (because no laboratory is involved). The major disadvantages of this method are the emergence of antimicrobial resistance (due to the use of antibiotics without laboratory conformation) and the high level of training required to perform SCM (18).

In this present study, we describe programme exposure, STI incidence rate, and condom use during follow-up. Furthermore, we aimed to explore whether subgroups of FSWs, as defined by age, soliciting place, and zone in Bangalore, showed differences in outcome measures.

## Methods

### The Pragati programme

The Pragati programme, which was launched in April 2005, was financially supported by the Bill and Melinda Gates Foundation, the Karnataka State AIDS Prevention Society (KSAPS), the United Nations Development Programme (UNDP), and Vrutti (a livelihood resource centre). The programme was implemented by Swathi Mahila Sangha (SMS), a sex worker collective, in collaboration with Swasti, a health resource centre. SMS managed field operations (particularly community mobilisation and outreach activities). Swasti was responsible for project and financial management, organisational development (of SMS), and technical support (e.g. strategy development, planning, monitoring, and evaluation). In addition, Swasti and its partner organisation Vrutti were involved in setting up and managing the Women's Bank (Swathi Jyothi), and they provided on-going technical support and capacity building.

During the Pragati programme design phase, SMS and Swasti consulted members of the sex work community to understand their needs. These needs were prioritised, and different services were designed to address these needs. Several community consultations continued throughout the Pragati programme, with a minimum of two consultations annually. In this way, the programme continues to remain relevant, and new needs are added to the Pragati programme when necessary. Sustainability was one of the key objectives of the Pragati programme. Therefore, Pragati was driven by the FSWs themselves. Pragati involved the FSW community at all stages, from conceptualisation and planning to implementation, with the community leaders involved in all decision-making forums (e.g. strategic planning and monthly review meetings). A shadow leadership approach (recognised by UNDP's ‘Capacity Is Development’ initiative as one of the top case studies) was adopted from the start of the programme, with the SMS staff shadowing the Swasti staff in all key positions. Swasti focussed on institutional strengthening of the SMS as well as building its leadership. SMS has grown from a membership of 13 in 2005 to 6,649 members in 2010 and has a decentralised leadership model with a central board and zonal boards (all democratically elected), which has helped ensure transparency, effective governance, and transition of leadership from those who founded SMS to the emerging second line of leadership.

The Pragati programme has facilitated the creation of a common platform for FSWs in Bangalore, and this platform works on issues common to all FSWs. The main goal of Pragati is to reduce HIV and STI transmission among FSWs and improve their well-being by developing their capacities. Pragati is categorised into three broad areas of intervention: (1) protect and respond, which involves implementing an outreach strategy in which peer educators and outreach workers start a dialogue with the FSWs about the services and benefits offered through the Pragati programme (this strategy also involves sensitising primary and secondary stakeholders of the sex worker industry, e.g. brothel owners, pimps, and the police, to the problems and needs of FSWs); (2) improve their quality of life through identifying and addressing long-term development needs such as providing support for alcohol de-addiction, providing savings and credit facilities, and creating options for alternative and diversified livelihoods; and (3) build the capacities of the FSWs to address the issues that threaten their lives and livelihoods through strengthening group action and developing a strong collective of FSWs.

### Empowerment strategy

The Pragati programme followed an empowerment approach (largely drawn from the work of Dr Srilatha Batliwala) which places the FSWs at the centre and seeks to address the internal and external factors that affect their vulnerability to HIV and STIs (19). The internal factors were the FSWs’ own beliefs, myths, and misconceptions; their low levels of confidence and capacity; their lack of ability to bargain collectively; and their unquestioning acceptance of established power and social structures. The external factors were the violence and harassment inflicted upon the FSWs, discrimination caused by social structures, and the legal framework within which the sex work industry operates. Taking into account the above factors, the programme set out to empower the women to (1) improve their ability to address their own well-being, (2) make informed choices, (3) improve access and control resources, and (4) realise their rights.

To achieve these overall outcomes, the Pragati programme focussed on ways to improve the FSWs’ self-consciousness and confidence, build their capacities, strengthen individual and collective action, and reduce the violence and harassment that are experienced by the FSWs. Pragati activities included capacity development, representation and democratisation, fostering leadership, creative advocacy, and alliance building with stakeholders.

### Pragati programme activities

The activities of the Pragati programme range from social and economic empowerment tools like crisis response teams (which try to diminish the amount of violence and harassment that FSWs face) to financial support by microfinance systems and health-related components such as de-addiction programmes, condom use promotion, and STI prevention and treatment strategies.

Services at the drop-in centres included provision of information and counselling on HIV and AIDS, demonstration of correct and consistent condom use, and free distribution of male and female condoms. FSWs can attend alcohol de-addiction programmes. Facilities for taking a bath, sleeping, and entertainment were present. Provision of relevant legal information, and savings and credit facilities through a microfinance institution established exclusively for FSWs, were available in the drop-in centres.

Data on services provided at the drop-in centres were recorded. When experiencing health problems, women could visit one of the participating clinics (programme-linked clinics or private referral clinics). Inside each drop-in centre, a clinic was organised where medical staff were present to provide health-related counselling, medical examinations, and treatment when needed based on SCM. An extensive overview of the services provided by the Pragati programme has been described elsewhere (17).

### Participants

All FSWs living and working in administrative zones 1, 3, 4, and 6 of Bangalore (estimated population: 9.6 million) were eligible for voluntary participation (20). From the start of Pragati in April 2005, FSWs were approached to consider participation in the programme by peer educators and outreach workers (all former or current FSWs). They motivated the FSWs to visit programme drop-in centres by explaining what services are provided there. Within the four programme zones of Bangalore, several drop-in centres were accessible to offer the FSWs a safe place to rest and relax, take a shower, have a meal, meet peers, and get in contact with the Pragati staff. During these contacts, FSWs were informed about the different services and areas of support available for them. Within the daily routine of the programme data on STIs, contacts and condom use were recorded. Upon registration, data on socio-economic status and sex work details of the participating FSWs were obtained. When experiencing health problems, the FSWs could visit one of the participating clinics (programme-linked clinics or private referral clinics). Inside each drop-in centre, a clinic was organised where medical staff were present to provide health-related counselling, medical examination, and treatment when needed. During these clinic visits, details including diagnosing and treating STIs, and data on the use of a condom at last paid sex were registered. Data on contacts with the Pragati employees, presence at a condom use demonstration, and the number of condoms distributed were also recorded.

### STI diagnosis

In both referral and programme clinics, STI diagnosis for FSWs was based on SCM and implied identification of consistent groups of symptoms (21, 22). Medical doctors were trained to use the SCM algorithm and provided immediate treatment for each reported syndrome. SCM contains algorithms for a broad range of symptoms. In the present analyses, we focus on the most common STI syndromes: genital ulcers, cervicitis, vaginitis, and PID.

### STI incidence rate

In line with the SCM protocol, all symptoms of the same STI syndrome, which were reported within 7 days of a previous visit (14 days for vaginitis), were considered as the same episode of that STI (21, 22). STI incidence rate was calculated as the sum of episodes for each specific STI syndrome divided by the sum of person-years.

### Programme exposure

The follow-up time of an FSW to the Pragati programme was calculated as the time between the date of the first contact and the date of the last contact with the programme, and it is expressed as person-years. The contact rate was defined as the total number of contacts of the FSWs within the programme (registration contacts, visits to drop-in centres, clinics, and contacts with peers and outreach workers) divided by the sum of person-years.

### Condom distribution

Data on condom distribution were used to estimate the total number of condoms dispensed per FSW per follow-up year. The condom distribution rate was calculated as the sum of condoms dispensed free of costs by the programme, divided by the sum of person-years. Additionally, the proportion of contacts during which one or more condoms were distributed and the proportion of FSWs who used a condom at the last paid sex were calculated.

### Statistical analysis

STI incidence rate, programme exposure (number of contacts per person-year), and condom distribution were analysed for the different years of follow-up. Because the Pragati programme concerns a dynamic cohort, the in- and outflow of the participating FSWs is not evenly distributed over the calendar years, as described earlier (17). We have analysed the data in such a way that each FSW starts on time point 0 independent from the year of entry in the programme, following the total exposure time. Additionally, the STI incidence rate, contact rate, and condom distribution over follow-up time were analysed and stratified by age, soliciting place, and geographical zone in Bangalore. Finally, the absolute number of diagnosed STIs per FSW who contributed person-time to a specific follow-up year was calculated. With these data, the proportion of FSWs without STI syndromes was calculated per follow-up year. Confidence intervals were calculated using the method of Rothman and Greenland (23). Trends over time were analysed with the Cochrane–Armitage test for trend (R Foundation for Statistical Computing, Vienna, Austria). All other analyses were performed with PASW Statistics 18 and SPSS version 18.0.

## Results

### Diagnosis of STIs within clinics

**Fig. 1:**
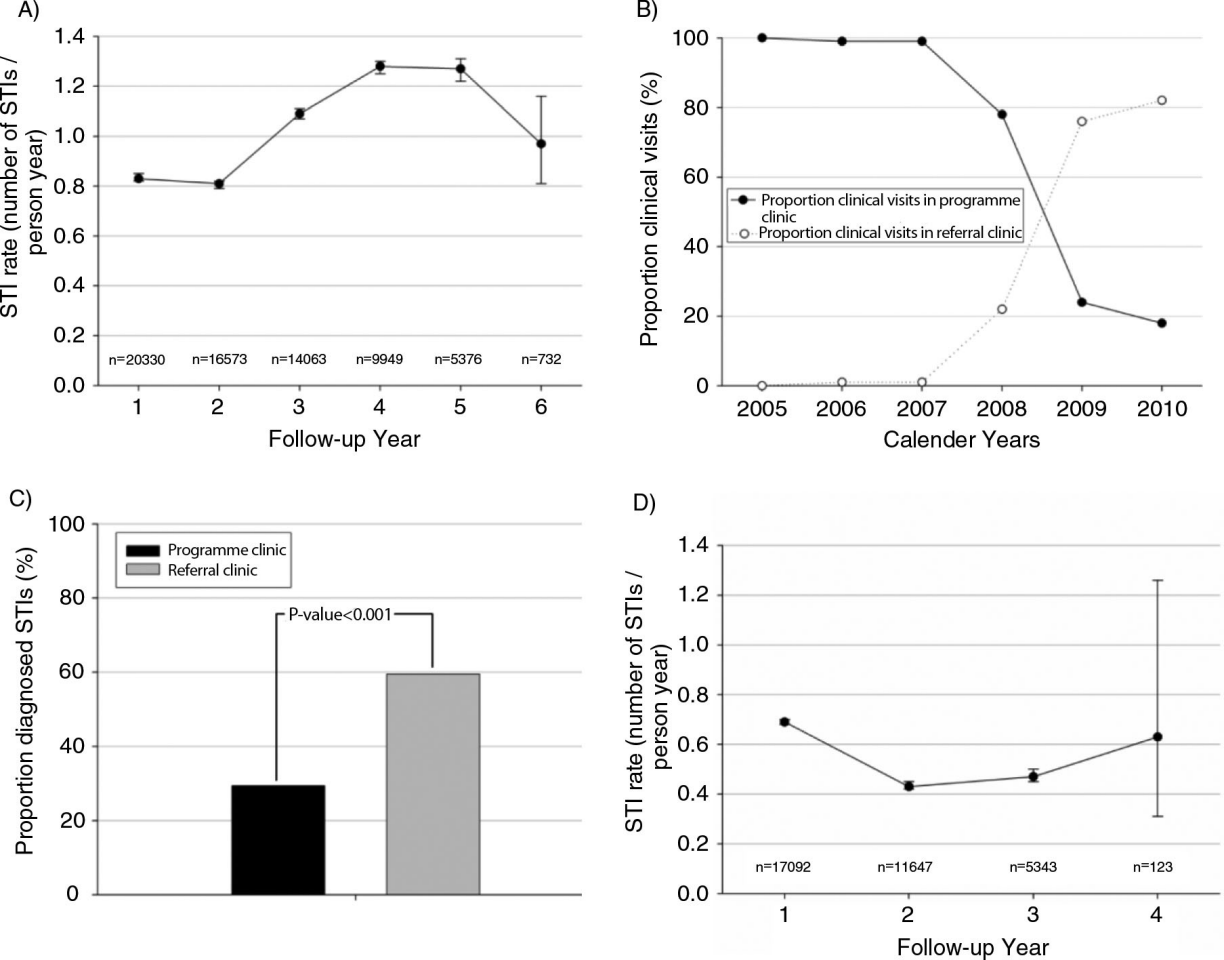
(A) The STI incidence rate over follow-up years between 2005 and 2010; (B) the proportion of clinical visits to the referral and programme clinics over calendar years between 2005 and 2010; (C) the proportion of visits during which an STI was diagnosed (STIs per clinic type); and (D) the STI incidence rate over follow-up years between 2005 and 2008.

Fig. 1A shows the STI incidence rate in relation to the programme exposure of the participating 20,330 FSWs (between April 2005 and November 2010). The incidence rate increased after follow-up year 2, indicating an adverse effect of exposure to the programme. However, the network of the participating clinics (programme-linked clinics or private referral clinics) in which the STIs were diagnosed and treated may well have influenced these data. In 2005, the Pragati programme started with one programme clinic, and this number increased to eight programme clinics in 2008. The number of contracted referral clinics increased from one clinic in 2005 to 90 clinics in 2008 (17). As a consequence, the number of referral clinic visits gradually started to outnumber the proportion of visits to programme clinics between 2005 and 2010 (Fig. 1B). Although STI diagnosis was based on the same protocol (SCM) in both clinic types, the proportion of visits during which an STI was diagnosed was significantly higher in referral clinics (59.5%) compared to programme clinics (29.4%) (chi-square test, *p*< 0.001) (Fig. 1C). These data suggest a discrepancy in the STI diagnoses that were performed within the two types of clinics. This discrepancy resulted in the increased STI incidence rate that is shown in Fig. 1A, which primarily seems to reflect the increased proportion of visits in referral clinics but not an actual increase in STI incidence. In an attempt to minimise the influence of the differences between programme and referral clinics with respect to STI diagnosis, we have chosen to restrict our analyses to the period between April 2005 and July 2008, when the majority of STIs were diagnosed in programme clinics (Fig. 1B). The systematic difference in STI diagnosis between clinic types could therefore not influence our analyses considerably.

### Study population

Between April 2005 and July 2008, 17,092 individual FSWs participated within the Pragati programme (Table 1). The median age (range) of the FSWs was 29 (10–60) years, and half of the FSWs were currently married (49.8%). Almost all (99.8%) FSWs had been working in the sex industry for longer than 2 years, and the majority (80.5%) reported having one or two clients per day on average. Most of the FSWs solicited either from home (37.5%) or in public areas (31.2%). Half (50.5%) of the FSWs were illiterate, and the predominant religion was Hinduism (78.6%). The mean (SD) follow-up time was 1.48 (0.84) years, resulting in a total of 25,369 person-years.

**Table 1:** Characteristics of participating female sex workers (FSWs) (*n*=17,092)

		Follow-up year			
		
Characteristics	Total	1	2	3	4
Number of FSWs	17,092	17,092	11,647	5,343	123
Follow-up time (years)	25369.2	14139.5	8962.1	2255.0	12.7
Median age, years (IQR)	29 (24–35)	29 (24–35)	29 (24–35)	29 (24–35)	30 (25–35)
Age group†					
10–23 years	3,961 (23.3%)	3,961 (23.3%)	2,612 (22.5%)	1,241 (23.4%)	27 (22.1%)
24–28 years	4,480 (26.3%)	4,480 (26.3%)	3,006 (26.0%)	1,380 (26.0%)	29 (23.8%)
29–34 years	3,488 (20.5%)	3,488 (20.5%)	2,392 (20.6%)	1,082 (20.4%)	30 (24.6%)
35+ years	5,081 (29.9%)	5,081 (29.9%)	3,586 (30.9%)	1,611 (30.2%)	36 (29.5%)
Number of clients per day§					
1	5,123 (37.8%)	5,123 (37.8%)	3,307 (36.6%)	1,436 (36.7%)	26 (47.3%)
2	5,781 (42.7%)	5,781 (42.7%)	4,016 (44.4%)	1,613 (41.1%)	19 (34.5%)
3 or more	2,648 (19.5%)	2,648 (19.5%)	1,720 (19.0%)	869 (22.2%)	10 (18.2%)
Soliciting place¶					
Home	5,508 (37.5%)	5,508 (37.5%)	3,256 (33.5%)	1,218 (28.4%)	9 (13.8%)
Rented room	2,544 (17.3%)	2,544 (17.3%)	2,020 (20.8%)	701 (16.3%)	13 (20.0%)
Lodge	767 (5.2%)	767 (5.2%)	591 (6.1%)	305 (7.1%)	9 (13.8%)
Brothel	734 (5.0%)	734 (5.0%)	446 (4.6%)	199 (4.6%)	1 (1.5%)
Street, park, or in public	4,576 (31.2%)	4,576 (31.2%)	2,974 (30.6%)	1,653 (38.5%)	28 (43.2%)
Other*	556 (3.8%)	556 (3.8%)	431 (4.4%)	218 (5.1%)	5 (7.7%)
Education‡					
Illiterate	7,612 (50.5%)	7,612 (50.5%)	5,141 (50.5%)	2,249 (47.0%)	75 (63.0%)
1–4 standard	1,637 (10.9%)	1,637 (10.9%)	1,057 (10.4%)	479 (10.0%)	8 (6.7%)
5–7 standard	2,642 (17.5%)	2,642 (17.5%)	1,820 (17.9%)	904 (18.9%)	15 (12.6%)
8–10 standard	2,576 (17.1%)	2,576 (17.1%)	1,772 (17.4%)	954 (20.0%)	17 (14.3%)
11 standard and above	528 (3.5%)	528 (3.5%)	363 (3.6%)	179 (3.8%)	4 (3.4%)
Literate but not been to school	69 (0.5%)	69 (0.5%)	29 (0.2%)	16 (0.3%)	0 (0%)
City zone					
Zone 1	3,909 (22.9%)	3,909 (22.9%)	2,801 (24.1%)	1,491 (27.9%)	9 (7.3%)
Zone 3	4,533 (26.5%)	4,533 (26.5%)	3,113 (26.7%)	1,963 (36.7%)	107 (87%)
Zone 4	4,034 (23.6%)	4,034 (23.6%)	2,689 (23.1%)	732 (13.7%)	1 (0.8%)
Zone 6	4,616 (27.0%)	4,616 (27.0%)	3,044 (26.1%)	1,157 (21.7%)	6 (4.9%)

Data represent numbers (%) unless indicated otherwise. IQR = interquartile range.

† Data were available for 17,010 women;

§ data were available for 13,552 women;

¶ data were available for 14,685 women;

‡ data were available for 15,064 women.

* Including FSWs soliciting from a *dhaba* (restaurant), bar or nightclub, vehicle, and theatre.

As the FSWs participating in the Pragati programme belong to a dynamic cohort, the decrease in participating FSWs per follow-up year does not reflect actual dropouts, but reflects mainly the in- and outflow of FSWs participating over the calendar years. Few FSWs started the programme in 2005 and are therefore exposed for the full 4 years. The 123 women participating in follow-up year 4 reflects only the women who started the programme in 2005. Sadly, no loss to follow-up data is known.

### Sexually transmitted infections

The STI incidence rate was 0.69 (95% confidence interval (CI): 0.68–0.70) STIs per person-year in the first year of follow-up, and it decreased significantly over time (*p*-value for trend < 0.001) (Fig. 1D). In years 2, 3, and 4, the STI incidence rates (95% CI) were, respectively, 0.43 (0.42–0.45), 0.47 (0.45–0.50), and 0.63 (0.31–1.26) STIs per person-year.

The supplementary file to this article shows the variety in recorded STIs per follow-up year. The majority of the 14,687 recorded STIs were either cervicitis (53.3%) or vaginitis (36.7%), while PID, genital ulcer non-herpes, and genital ulcers comprised 9.8% of the total number of recorded STIs.

**Fig. 2:**
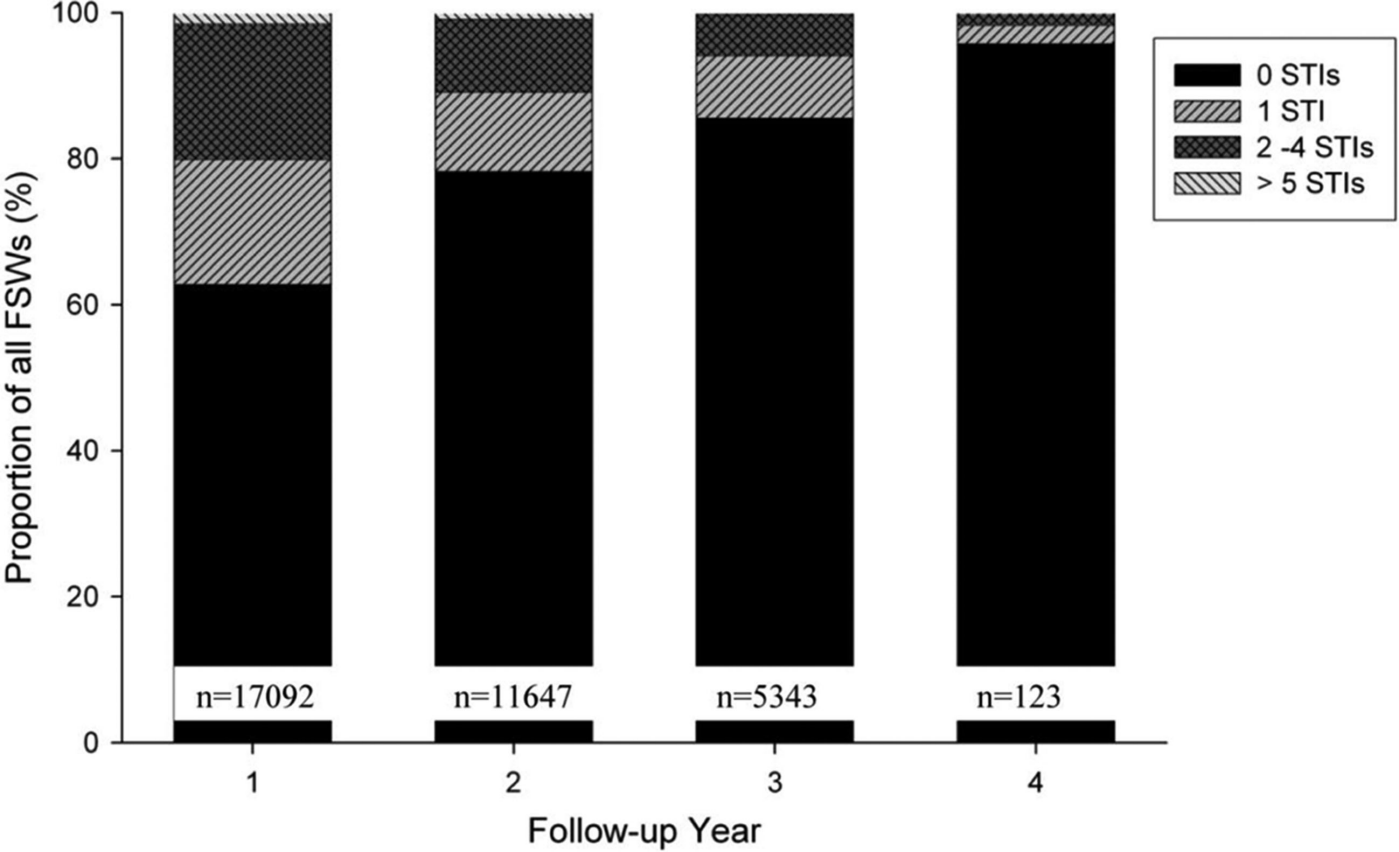
Proportion of FSWs without STIs or with 1, 2–4, or more than 5 STIs over follow-up time. Bars represent the proportion of all FSWs divided into four groups (1: no STI, 2: 1 STI, 3: 2–4 STIs; and 4: more than 5 STIs).

When analysing the proportion of FSWs by increasing number of STIs recorded (0, 1, 2–4, or 5 or more STIs) per follow-up year, the data showed that the proportion of FSWs without STIs significantly increased from 63% in follow-up year 1 to 96% in year 4 (*p*-value for trend < 0.001) (Fig. 2).

In general, with the exception of a few subcategories, the STI incidence rate showed a decrease over time (Supplementary file).

### Programme exposure

The contact rate of FSWs in the first year of follow-up was 9.49 (95% CI: 9.44–9.54) contacts per person-year, and it increased significantly over time (*p*-value for trend = 0.007) (Supplementary file). The contact rates (95% CI) in year 2, 3, and 4 of follow-up were, respectively, 8.40 (8.34–8.46), 11.65 (11.51–11.80), and 9.12 (7.60–10.94) contacts per person-year.

When analysing subgroups (for age, soliciting place, and zone in Bangalore), all showed an increase in contact rate over time (Supplementary file). There were no major differences in contact rates between different age groups. With respect to the soliciting place, those FSWs soliciting from rented rooms had the lowest contact rate, 7.89 (CI: 7.80–7.97) contacts per person-year, whereas those soliciting from ‘other’ places (restaurants, bars, night clubs, vehicles, and theatres) had the highest contact rate: 11.56 (CI: 11.34–11.78) contacts per person-year.

### Condom distribution

**Fig. 3:**
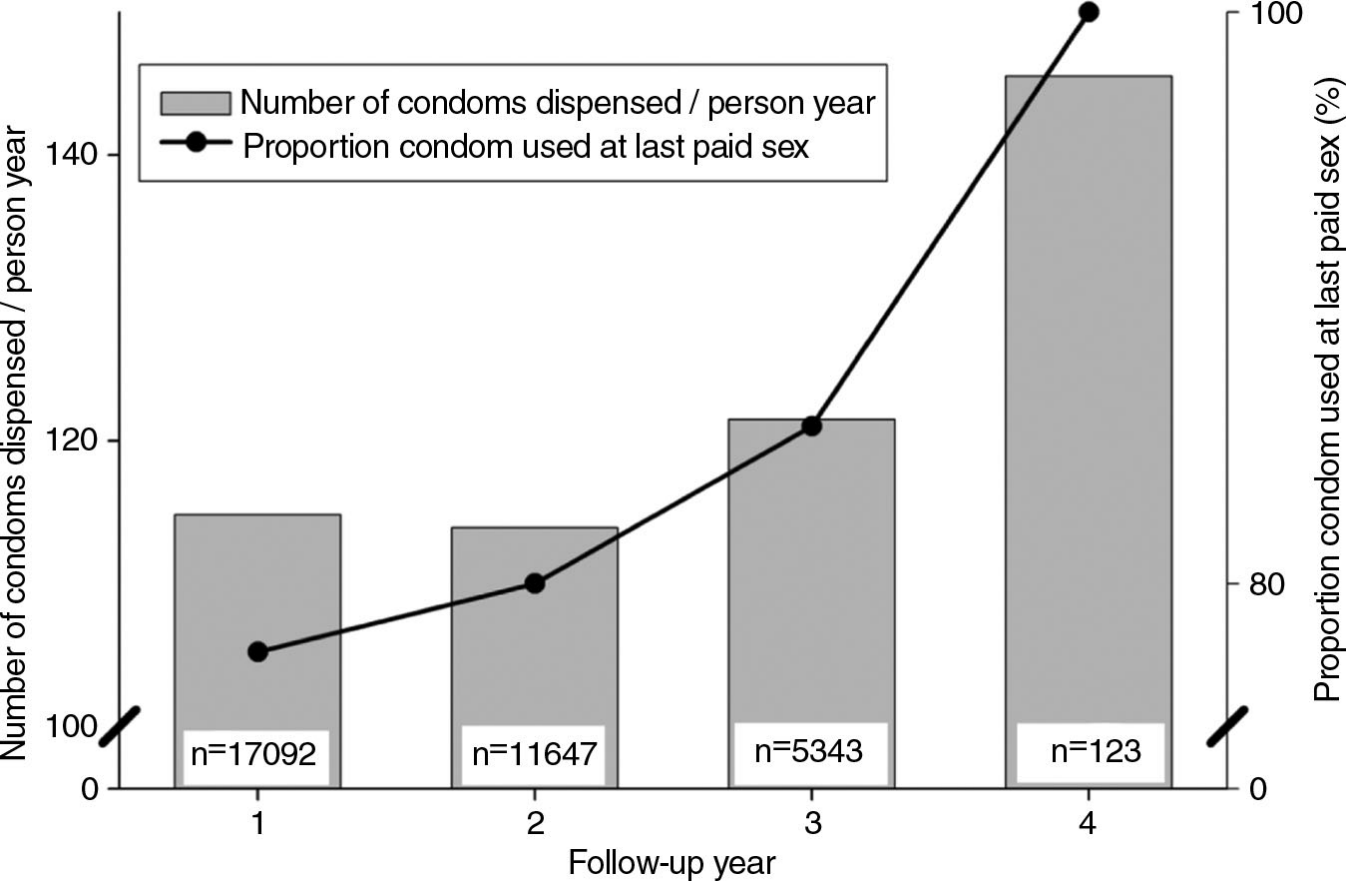
Condom distribution and use over follow-up time. Black dots represent the proportion of FSWs who had used a condom at last paid sex. The bars represent the number of condoms dispensed per person-year. Please note that the y-axis on the left ranges from 100 to 150 condoms dispensed per person-year; the y-axis on the right ranges from 75 to 100% of FSWs who used a condom at last paid sex.

The number of condoms dispensed per person-year was 114.8 (CI: 114.6–115.0) condoms in year 1, and this increased over time, although not significantly (*p*-value for trend = 0.112) (Fig. 3). The number of condoms dispensed per person-year in year 2 was 113.9 (CI: 113.7–114.1), in year 3 it was 121.5 (CI: 121.1–122.0), and it was 145.5 (CI: 139.0–152.3) condoms per person-year in year 4. Stratified analysis showed no large differences between subgroups (Supplementary file). The proportion of FSWs who used a condom at the last paid sex increased from 77.6% in year 1, to 85.5% in year 3, and to 100% during the fourth year of follow-up (although this was based on a relatively low number of participating FSWs, *n*=123) (Fig. 3) (*p*-value for trend < 0.001). Additionally, the proportion of contacts with the programme during which condoms were dispensed increased from 40.3% in year 1 to 54.3% in year 4 (*p*-value for trend < 0.001).

## Discussion

Our study shows that the FSWs participating in the Pragati programme had a significant increase of programme exposure, used more condoms, and showed a significant decrease in STI incidence rate during a relatively short follow-up period. Not only the number of recorded STIs per person-year decreased, but the proportion of FSWs with one or more STI per person-year also decreased during follow-up. Although this may suggest that the activities of the empowerment strategy in the Pragati programme have affected the STI incidence rate, it should be noted that the absence of an adequate control group restricts the interpretation of these findings.

The Pragati programme allowed us to analyse longitudinal data from a large number of participating FSWs for whom all contacts with the programme, STIs, and condom usage were recorded. Our data on programme exposure showed an increasing trend, indicating a higher contact rate for those FSWs who were exposed to the Pragati programme for a longer time. Due to a still relatively short follow-up time and the limited number of participating women in year 4, these results should be interpreted with some caution. Unfortunately, the structure of the Pragati programme, in which STIs are diagnosed during visits to clinics that are an integral part of the programme, limited the possibility to analyse the available data in a case–control or single-cohort design. Within the participants of the Pragati programme, it was not possible to select a control group for whom STI diagnosis during the study period was made independently of contacts (exposure) with the programme. The availability of such a control group would have provided the possibility to quantify the results of the Pragati programme more clearly, and we would therefore suggest the incorporation of a control group in the study design for intervention programmes that aim to evaluate the results of their intervention after implementation

Previous other studies showed an increase in contacts with the programme over time, which is in line with our data (11, 12). However, most previous studies were cross-sectional surveys and did not follow FSWs prospectively during the study period (24). Several studies reported reduced STI and HIV incidence rates as an effect of an intervention, but these studies showed large differences in study design, intervention strategy, and study population (11, 25–27). Wariki et al. showed favourable results for behavioural change interventions above single interventions. A novel aspect of the Pragati programme is that it uses an empowerment strategy within its intervention efforts. As hypothesised before, the empowerment strategy could improve programme results in addition to standard programme interventions (e.g. condom use promotion and STI services).

Furthermore, in contrast with Pragati, most studies reported STIs confirmed with laboratory tests. In the present study, we report STI syndromes based on SCM. Bosu has placed emphasis on the use of SCM instead of laboratory tests, especially in poor-resource countries where SCM could have major advantages (18). He stated that SCM is cost-effective in relation to laboratory tests and that SCM has better compliance since diagnosis and treatment are both provided at the first visit (18). Other advantages are a high cure rate, rapid treatment (because no laboratory conformation is needed), and easy integration into management and programme systems (26). Reported sensitivity and specificity of SCM are high, and they are mostly greater than 80% except for the detection of *Trichomonas vaginalis*, herpes simplex virus-2 (HSV2), and *Candida albicans* (28, 29). Disadvantages of SCM are overtreatment in cases of vaginal discharge where cervicitis is not the predominant cause. These FSWs are then unnecessarily exposed to antibiotics, which could result in the emergence of antimicrobial resistance (30). Another disadvantage is missing asymptomatic cases (because SCM only detects symptomatic cases); therefore, SCM (to diagnose STIs) could never completely stop transmission. However, in poor-recourse countries where laboratory facilities are unavailable, the advantages seem to outweigh the disadvantages.

An important component of the Pragati programme is the promotion of condom use. Our data show that the proportion of FSWs who reported using a condom at last paid sex, the number of condoms dispensed per person-year, and the proportion of contacts with the programme at which condoms were distributed increased between year 1 and year 4 of follow-up. These findings are in line with other studies (12, 31). Following stratification for age, soliciting place, and zone in Bangalore, similar patterns were seen.

The present study has some limitations. First, data on STIs, contacts with the programme, and condom use were collected within the daily routine of the Pragati programme. This entailed various problems; for instance, visits to clinics were an integrated part of exposure to the programme. Therefore, data on the outcome variable (STI incidence) were not independently recorded but were related to the exposure variable (contacts with the programme). Second, there was a relatively short mean follow-up time (1.48 years), which may have limited the possibility to address the effects of the programme. Third, this study lacked a control group, and it was therefore difficult to clearly differentiate the effects of the Pragati programme on the outcome measurements from the changes that have occurred independently of Pragati. Fourth, STI diagnosis based on SCM gave us no laboratory confirmation of the cause of infection. Fifth, the discrepancy in the diagnosis of STIs between programme clinics and referral clinics complicated the analyses that were restricted to the period between April 2005 and July 2008. The percentage of clinical visits that resulted in a diagnosis of an STI was significantly higher in referral clinics, which forced us to restrict our analyses. There are several possible explanations for this discrepancy: The level of training of medical doctors who performed SCM may have differed between programme clinics and referral clinics. Furthermore, the medical staff in the referral clinics were financially incentivised for each STI diagnosis made.

In line with other studies, we recommend for future studies to invest more in the study design, type of data collection, and recording mechanisms (the outcome measure must be recorded independently from the exposure) before starting an intervention (12, 24). Intervention programmes must be set up with the possibility to evaluate the results annually. For example, longitudinal data could be analysed with a case–control or single-cohort design. In addition, once a year, all participating FSWs could be screened with laboratory-confirmed STI tests to determine the true prevalence.

In conclusion, we were able to describe condom use, programme exposure, and STI incidence rate over follow-up years for the participating FSWs in the Pragati programme. These FSWs showed a significant increase in programme exposure and condom use and a significant decrease in STI incidence rate. It will be interesting to report on a longer follow-up period to see how the STI incidence rate develops, ideally with the incorporation of a control group, to further quantity the results of the Pragati empowerment programme on the lives and livelihoods of the participating FSWs in Bangalore.
